# Simulated microgravity attenuates myogenic differentiation via epigenetic regulations

**DOI:** 10.1038/s41526-018-0045-0

**Published:** 2018-05-23

**Authors:** Takuma Furukawa, Keiji Tanimoto, Takahiro Fukazawa, Takeshi Imura, Yumi Kawahara, Louis Yuge

**Affiliations:** 1 Division of Bio-Environmental Adaptation Sciences, Graduate School of Biomedical and Health Sciences, Hiroshima University, Hiroshima, Japan; 2 Department of Radiation Medicine, Research Institute for Radiation Biology and Medicine, Hiroshima University, Hiroshima, Japan; 3 Natural Science Center for Basic Research and Development, Hiroshima University, Hiroshima, Japan; 4 Space Bio-Laboratories Co., Ltd, Hiroshima, Japan

## Abstract

The molecular mechanisms involved in myogenic differentiation are relatively well-known. Myogenic differentiation is regulated by the sequential activation of the basic helix-loop-helix myogenic regulatory transcription factors (MRFs), and biomechanical signals play an important role in the regulation of myogenesis. In this study, we sought to determine whether simulated microgravity culture using Gravite^®^ may affect myoblast differentiation and expression of MRF genes. Although rat myoblasts, L6 cells were differentiated to myotubes in an incubation period-dependent manner, myogenesis of L6 cells was significantly attenuated under simulated microgravity (10^-3^G) conditions. Real-time Reverse transcription polymerase chain reaction (RT-PCR) showed that expressions of *Myog*, *Myf6*, *Mef2c*, *Des*, and *Ckm* under 1 G conditions increase in an incubation period-dependent manner, and that *Myod1* expression was specifically observed to increase transiently in the early phase. However, expressions of *Myod1* and *Myog* were significantly inhibited under simulated microgravity conditions. To clarify the molecular mechanisms, L6 cells were treated with 5-AzaC, and further incubated with differentiation medium under 1 G or 10^−3^ G conditions. The results showed differences in expression levels of *Myod1*, *Myog*, and, as well as those of myotube thickness between 1 G and 10^−3^ G conditions, completely disappeared in this experimental condition. Modified HpaII tiny fragment enrichment by ligation-mediated PCR (HELP)-assay showed that kinetic changes of DNA methylation status were attenuated in simulated microgravity conditions. These results indicate that microgravity regulates myogenesis and *Myod1* expression by controlling DNA methylation.

## Muscles: why does microgravity slow muscle fiber formation?

Muscle development is impaired under simulated microgravity because of alterations in the epigenetic regulation of critical muscle differentiation genes. Louis Yuge and colleagues from Hiroshima University, Japan, cultured rat muscle progenitor cells known as myoblasts under normal gravity and microgravity conditions, both with and without a drug that inhibits an enzyme involved in adding methyl tags to DNA to control gene expression. In the absence of this drug, microgravity-incubated cells formed muscle fibers at a slower rate and showed deficits in expression of two key differentiation genes. With the drug, however, these differences disappeared. The findings suggest that microgravity affects patterns of DNA methylation linked to regulating muscle formation. A similar process may be happening in the bodies of people with age-related muscle loss and weakness—highlighting a potential therapeutic target for future drug development.

## Introduction

Sarcopenia is defined as an age-related loss of skeletal muscle mass and strength. Beginning with the 4th decade of life, and symptoms progress with age.^1^ If muscle mass accounts for up to 60% of body weight, pathological changes in skeletal muscle can cause serious effects on older adults. Nevertheless, the therapeutic outcome of age-related skeletal muscle atrophy and weakness remains unknown.^2–4^ The understanding of molecular mechanisms of myogenic differentiation process will result in better treatment outcomes, since impaired regulation of myogenic differentiation is closely associated with age-related skeletal muscle dysfunction.^5–8^ It is well known that myogenic differentiation is regulated by the sequential activation of the basic helix-loop-helix myogenic regulatory transcription factors (MRFs): MyoD, Myf5, myogenin, and MRF4 (Myf6).^9–11^ MyoD in particular, is involved in the commitment of cells to the myogenic lineage.^12–14^ It has been reported that exposure of myoblast or muscle satellite cells to mechanical uniaxial stretch and stretching by a magnetic field, or electrical stimulation, upregulate MRFs expression, resulting in activation of myogenesis.^15–17^ Moreover, differentiation of myoblasts was enhanced on highly aligned fullerene whiskers scaffolds culture.^18^ In contrast, differentiation of myoblast was suppressed in the microgravity culture for in vitro study and spaceflight experiments, suggesting that biomechanical signals play an important role in the regulation of myogenesis.^19,20^ It is significant that the muscle mass of astronauts after 2 weeks space flight was diminished by up to 20%.^21^

DNA methylation is also known to be involved in regulation of myogenic gene expression and myogenesis: The DNA-demethylating agent 5-azacytidine induced myogenesis via upregulated MyoD expression.^22–25^ Barrès et al. interestingly reported that acute exercise tends to reduce global methylation on the entire genome, resulting in activation of regulated genes in human skeletal muscle.^26^ Singh et al. demonstrated that simulated microgravity induced epigenetic changes of genome DNA in human lymphocytes through altered expression of *DNMT1*, *DNMT3a*, and *DNMT3b*.^27^ However, the effects of space flight or simulated microgravity on epigenetic changes of MRF genes and resulting myogenesis remain unclear. Therefore, this study aims to determine whether simulated microgravity affects myoblast differentiation, expression of MRF genes, and the status of DNA methylation.

## Results

### Morphological evaluation of myogenesis under microgravity conditions

**Fig. 1:**
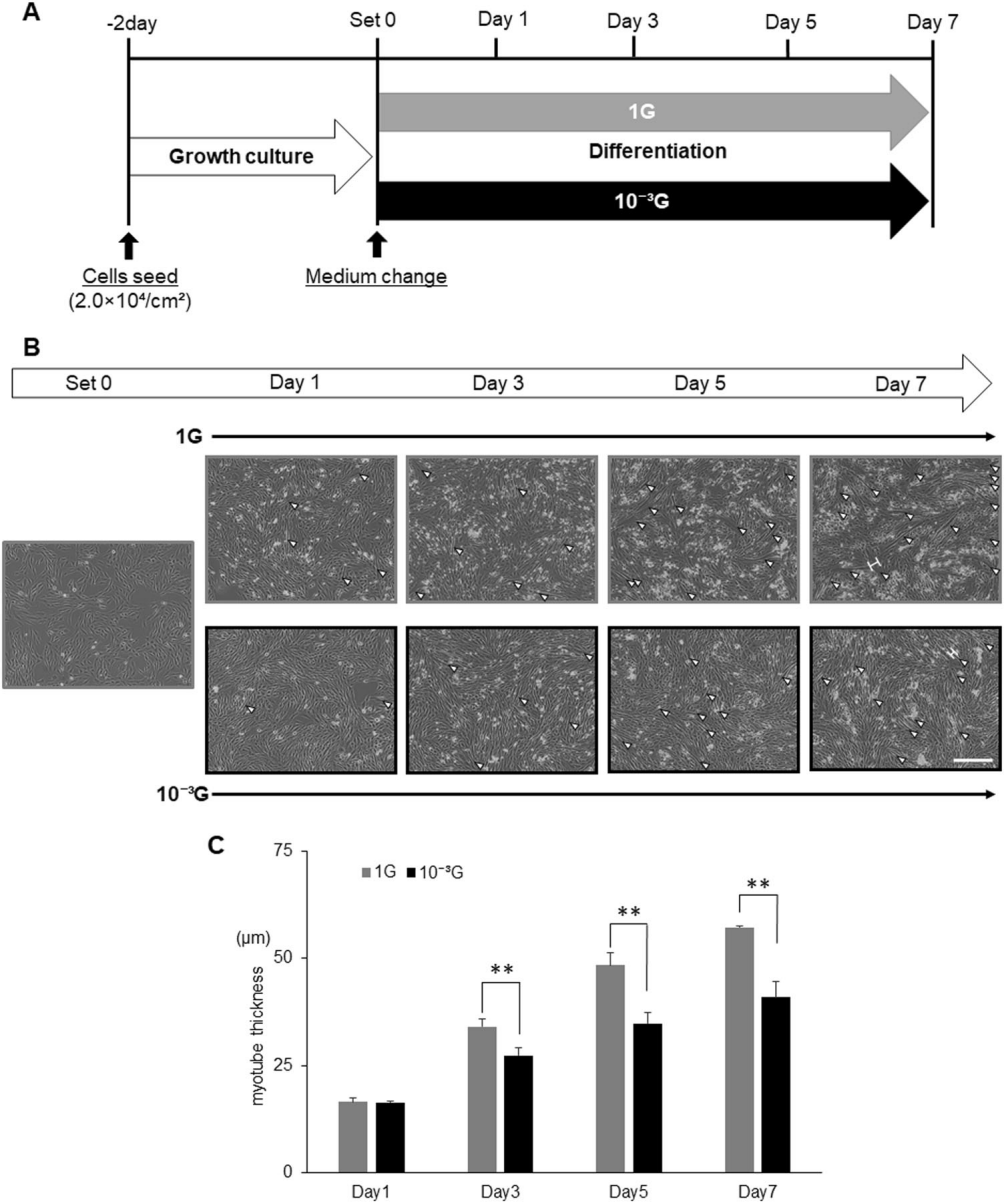
Morphological evaluation of myogenesis under microgravity conditions. **a** Schematic representation of experimental time course is shown. L6 cells were cultured with growth medium for 2 days, and then replaced with differentiation medium. Cells were then cultured under normal 1 G or 10^-3^G conditions for 0, 12 h, 1, 3, 5, or 7 days, and observed by taking pictures, and the maximum transverse diameters of myotube cells (myotube thickness) were measured. **b** Representative microscopic images during myogenesis are shown. White arrow heads: myotube cell; white H-shaped bars: examples of the maximum transverse diameter of myotube cells; white scale bar: 300 μm. **c** The maximum transverse diameters of myotube cells were measured by image processing software ImageJ, and the mean value of 10 fields was calculated. Columns show the mean of three independent experiments; bars, SD. *P* values calculated with Student’s *t* test. (**P < 0.01) (*n* = 3)

To evaluate myogenesis under simulated microgravity conditions, L6 cells were cultured with differentiation medium under normal 1 G or microgravity (10^-3^G) conditions for 1, 3, 5, or 7 days (Fig. 1a). Cells were then observed by capturing images, and the maximum transverse diameters of myotube cells (myotube thickness) were measured as indicated in Fig. 1b. As expected, L6 cells were differentiated to myotubes, resulting in an increase in the thickness, under 1 G conditions in an incubation period-dependent manner (Fig. 1c). Interestingly, myogenesis was significantly attenuated under microgravity conditions on day 3, 5, and 7, although myotubes gradually increased (Fig. 1c).

### Gene expression analyses of myogenesis-relating genes under microgravity conditions

**Fig. 2:**
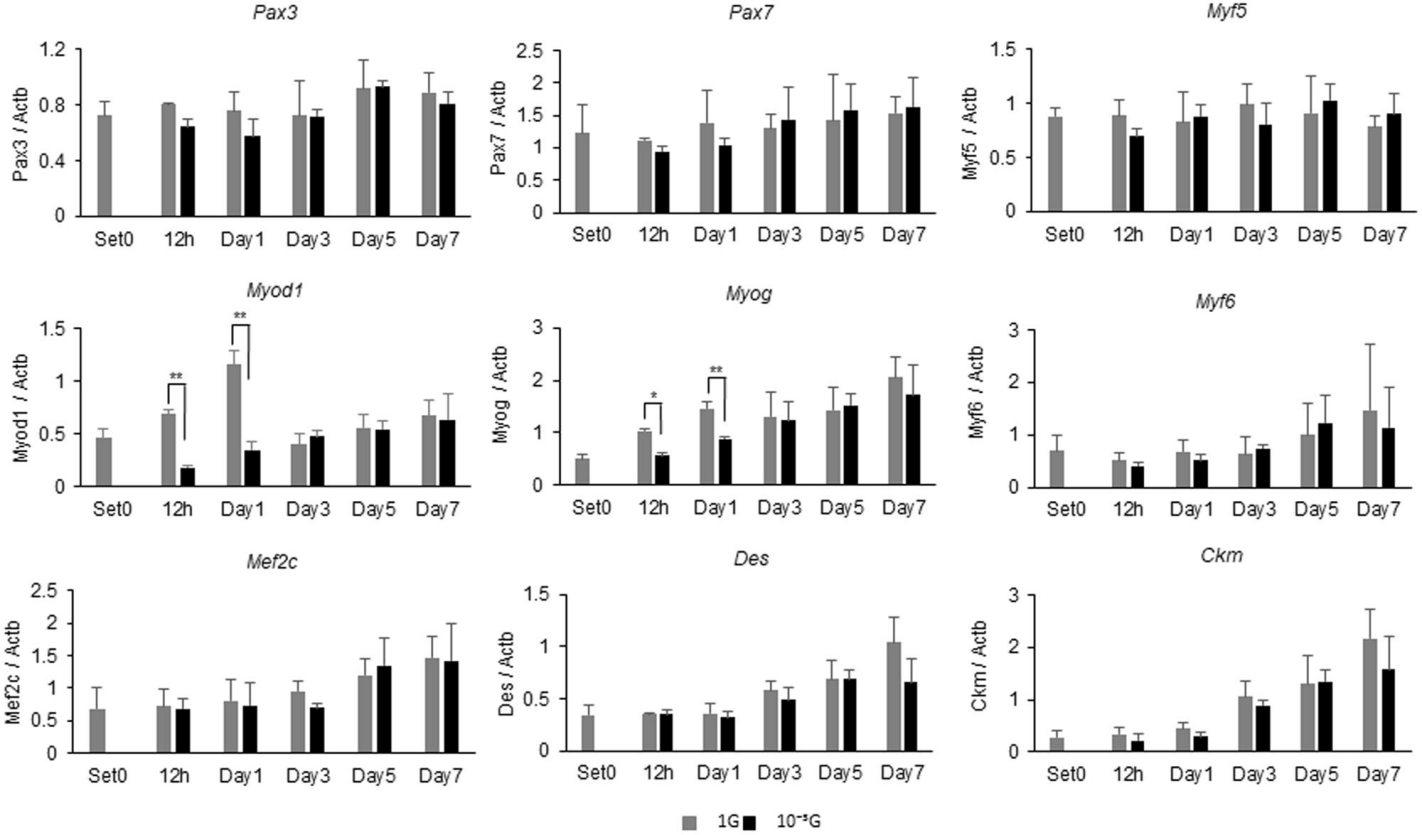
Gene expression analyses of myogenesis-related genes under microgravity conditions. For expression analysis, L6 cells were cultured under normal 1 G or 10^-3^G conditions for 0, 12 h, 1, 3, 5, or 7 days. Total RNA was extracted from cell pellets, and expression levels of myogenesis-relating genes were evaluated by real-time RT-PCR. Three independent measurements were averaged, and relative gene expression levels were calculated as a ratio against *Actb* expression for each experiment. Columns show the mean of three independent experiments; bars, SD. *P* values calculated with Student’s *t* test. (*P < 0.05, **P < 0.01) (n = 3)

In order to clarify the molecular mechanisms of attenuation of myogenesis under microgravity conditions, expression levels of myogenesis-related genes were evaluated using real-time RT-PCR (Fig. 2): Expression of *Myog*, *Myf6*, *Mef2c*, *Des*, and *Ckm* under 1 G conditions increased in an incubation period-dependent manner. Although transient increases of *Myod1* expression under 1 G conditions were observed from 12 h to Day1, those increases were significantly inhibited in microgravity conditions. *Myog* expressions were also significantly inhibited in microgravity conditions at the same points of time. Expression levels of *Pax3*, *Pax7*, and *Myf5* did not change under our study conditions.

### Effects of 5-Azacytidine treatment on myogenesis under microgravity conditions

**Fig. 3:**
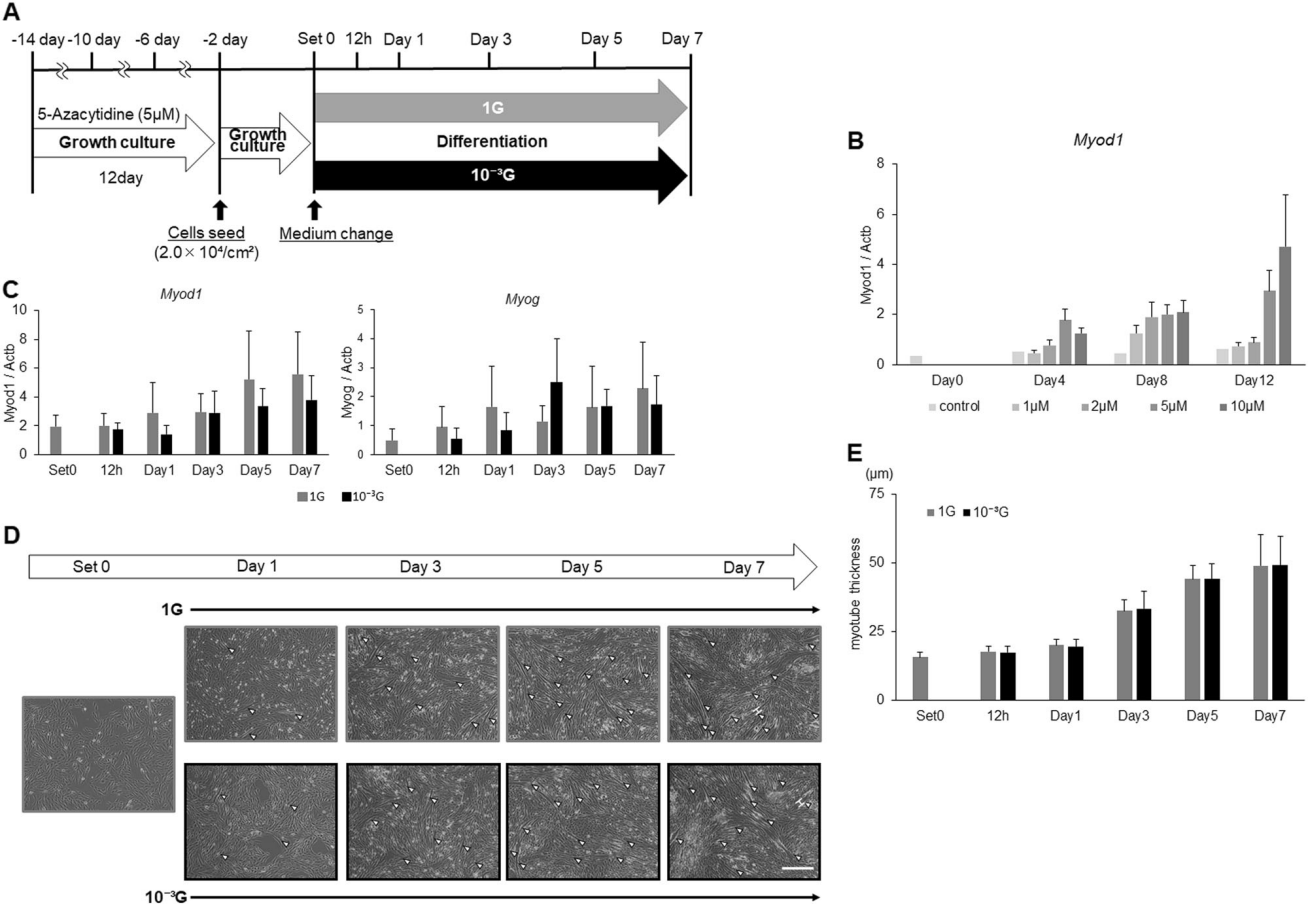
Effects of 5-Azacytidine treatment on myogenesis under microgravity conditions. **a** Schematic representation of experimental time course is shown. L6 cells were cultured with 5 μM of 5-AzaC in growth medium for 12 days, with passage every 4 days. Cells were then seeded on culture flasks and cultured in growth medium for 2 days. After medium replacement with differentiation medium, cells were cultured in normal 1 G or 10^−3^ G conditions for 0, 12 h, 1, 3, 5, and 7 days. **b** Optimization of 5-azaC treatment was performed. L6 cells were treated with 1, 2, 5, or 10 μM of 5-AzaC for 4, 8, or 12 days, and expression levels of *Myod1* gene were evaluated by real-time RT-PCR. Three independent measurements were averaged and relative gene expression levels were calculated as a ratio against *Actb* expression for each experiment. Columns show the mean of three independent experiments; bars, SD (*n* = 3). **c** Expression levels of *Myod1* and *Myog* genes were evaluated by real-time RT-PCR. Three independent measurements were averaged and relative gene expression levels were calculated as a ratio against *Actb* expression for each experiment. Columns show the mean of three independent experiments; bars, SD (*n* = 3). **d** Representative microscopic images during myogenesis are also shown. White arrow heads: myotube cell; white H-shaped bars: examples of the maximum transverse diameter of myotube cells; white scale bar: 300 μm. **e** The maximum transverse diameters of myotube cells were measured using image processing software ImageJ, and the mean value of 10 fields was calculated. Columns show the mean of three independent experiments; bars, SD. (*n* = 3)

Expression of *Myod1* and *Myog* had previously been reported to be regulated by DNA methylation.^23,25,28^ In order to clarify the molecular mechanisms of the decreased expression of *Myod1* and *Myog*, and the significance in myogenesis under microgravity conditions, L6 cells were then treated with DNA methylation inhibitor, 5-Azacytidine (5-AzaC), before incubation with differentiation medium (Fig. 3a). To optimize concentration and treatment periods of 5-AzaC, L6 cells were first treated with 1, 2, 5, or 10 μΜ of 5-AzaC for 4, 8, or 12 days. As a result, *Myod1* expression was observed to increase in a concentration- or treatment period-dependent manner (Fig. 3b). Of these conditions, treatment with 5 μΜ of 5-AzaC for 12 days was confirmed as the effective condition for the following experiments. After treatment with 5 μΜ of 5-AzaC for 12 days, L6 cells were incubated with growth medium for 2 days, and also with differentiation medium for the indicated periods under 1 G or microgravity conditions. Real-time RT-PCR showed that the expression levels of *Myod1* and *Myog* seemed to increase gradually in a treatment period-dependent manner, but the difference between 1 G and 10^−3^ G conditions observed in Fig. 2, and disappeared in Fig. 3c. In this experimental condition, morphological evaluations were also performed as in Fig. 1. Results showed that differences in myotube thickness between 1 G and microgravity conditions also completely disappeared, although L6 cells were gradually differentiated to myotubes under both conditions (Fig. 3d,e).

### DNA methylation in *Myod1* gene under microgravity conditions

**Fig. 4:**
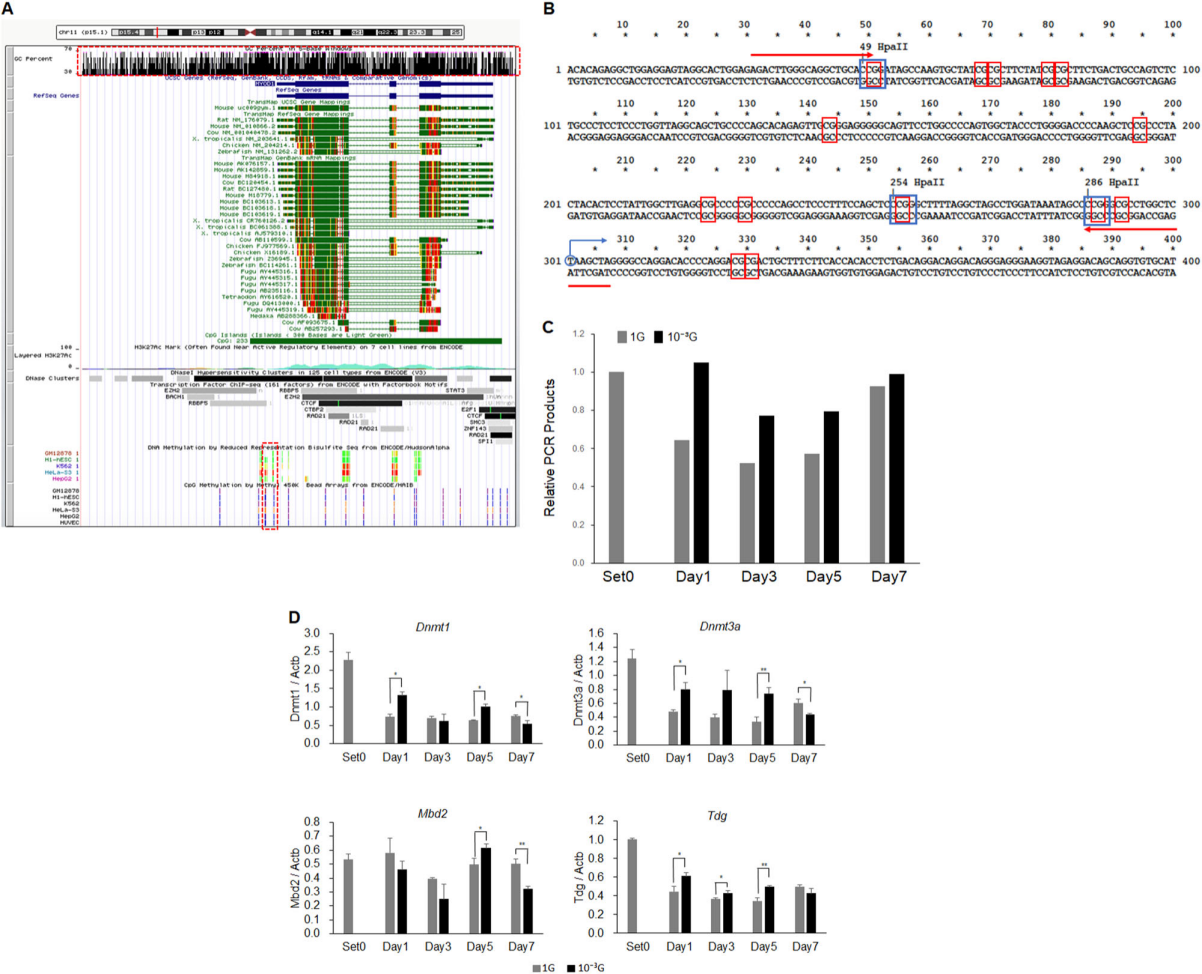
DNA methylation in *Myod1* gene under microgravity conditions. **a** Schematic structure of *MYOD1* gene in UCSC Genome Browser showed that *MYOD1* gene virtually overlapped with CpG island (upper rectangle drawn in red dotted line), indicating very dense CpG sites, and the proximal promoter region contained actually methylated sites (lower rectangle drawn in red dotted line). **b** The nucleic acid sequence of proximal promoter region in *Myod1* gene is shown. Blue circle and arrow indicate transcription start site of *Myod1* gene, red rectangles, CpG sites, blue rectangles, *Hpa*II sites, and red arrows, primer sets for modified HELP-assay. **c** Methylated proximal promoter regions in *Myod1* gene are quantified by real-time PCR. Three independent measurements were averaged and relative amounts of PCR products were calculated as the ratio against that of Set0 for each experiment. (*n* = 3). **d** Expression levels of methylation related genes were evaluated by real-time RT-PCR. Three independent measurements were averaged and relative gene expression levels were calculated as a ratio against *Actb* expression for each experiment. Columns show the mean of three independent experiments; bars, SD. *P* values calculated with Student’s *t* test. (*P < 0.05, **P < 0.01) (*n* = 3)

To determine whether DNA methylation actually contributes to regulation of *Myod1* expression, we use modified HELP-assay to detect DNA methylation in the promoter region of *Myod1* gene.^29^ UCSC Genome Browser (http://genome.ucsc.edu) showed that *MYOD1* gene virtually overlapped with CpG island, indicating very dense CpG sites, and the proximal promoter region contained actually methylated as shown in the results of reduced representation bisulfite sequencing and methyl 450 K bead arrays (Fig. 4a). A primer set was designed for just upstream from the transcription start site of *Myod1*, which contained 12 CpG or 3 *Hpa*II sites (Fig. 4b). Since DNA methylation sensitive restriction enzyme *Hpa*II cannot digest methylated DNA, but/although can digest unmethylated DNA, the primer set amplified methylated DNA, but not unmethylated or digested DNA after incubation with *Hpa*II. Therefore, methylated DNA can be quantified by real-time PCR, resulting in evaluation of DNA methylation status. Genomic DNAs were then isolated from L6 cell samples that had been cultured as in Fig. 1, and incubated with *Hpa*II for 12 h. Real-time PCR demonstrated that relative amounts of PCR products amplified from 1 G samples decreased gradually until Day3, and then increased until Day7 (Fig. 4c). It is striking that PCR products from microgravity samples also slightly decreased but to a smaller degree than those of 1 G, suggesting a retaining DNA methylation on *Myod1* promoter (Fig. 4c). In order to clarify the underlying mechanisms, real-time RT-PCR analyses of methylation-related genes were performed using the same samples of cDNA as in Fig. 2. Results showed that expressions of DNA methyltransferase, *Dnmt1* and *Dnmt3a*, under 1 G conditions were decreased during the myogenic process (Fig. 4d), while *Dnmt3b* was not detected (data not shown). Interestingly, expressions of *Dnmt1* and *Dnmt3a* under microgravity conditions also decreased during the myogenic process, but remained significantly higher than those under 1 G conditions, suggesting they retained functions of methyltransferases (Fig. 4d). Expression levels of *Tdg* also decreased during the myogenic process, but differences between 1 G and microgravity conditions were smaller. Expression levels of *Mbd2* varied and there was no trend observed.

### Other regulation of *Myod1* and *Myog* genes under microgravity conditions

In order to clarify regulation mechanisms of expression of *Myod1* and *Myog* genes during the myogenic process under 1 G and microgravity conditions, further analyses of promoter activities and RNA stabilities were performed. First, the 1.47 kb DNA fragment of the *Myod1* gene promoter region, or the 1.55 kb DNA fragment of the *Myog* gene promoter region, was amplified and subcloned into the luciferase reporter plasmid pGL4.16 (designated pGL4.16-Myod1 Pro1474 or pGL4.16-Myog Pro1546, Supplementary Fig 1B). L6 cells were then transfected with these promoter reporters and incubated for 1 day. Next, growth medium was replaced with differentiation medium and incubated for one more day under 1 G or microgravity conditions (Supplementary Fig 1A). Dual-luciferase assays demonstrated that cloned regions had strong promoter activities compared to empty vector, but any differences in those activities under 1 G or microgravity conditions were not observed (Supplementary Fig 1C). L6 cells were next cultured in growth medium for 2 days, and then cultured in differentiation medium for further a day. After adding 5 μg/ml of actinomycin D, cells were cultured under 1 G or microgravity conditions (Supplementary Fig 2A). Real-time RT-PCR showed that gene expression level of *Myod1* had decreased gradually, while those of *Myog* and *Actb* had not (Supplementary Fig 2B). There were no differences in those levels under 1 G or microgravity conditions (Supplementary Fig 2B).

## Discussion

As a consequence of an aging population, the number of elderly who exhibit skeletal muscle atrophy and weakness are increasing, becoming a concern of society.^30–32^ In order to achieve better treatment outcomes, the understanding of the molecular mechanisms of myogenesis is essential, since impaired myogenesis is closely associated with age-related skeletal muscle loss and weakness.^5–8^ Previous reports have suggested that biomechanical signals play an important role in the regulation of myogenesis. Specifically, exposure of myoblast and muscle satellite cells to mechanical stretching, electrical stimulation, or microgravity have been found to modify expression levels of MRFs, resulting in alteration of myogenesis.^15–17,19^

In this study, we used our new generation 3D-clinostat “Gravite^®^” to obtain simulated microgravity conditions (10^-3^G) for cell culture: We observed that myogenesis of rat myoblast cells (L6 cells) cultured in Gravite^®^ were significantly attenuated, indicating successful creation of microgravity conditions (Fig. 1). L6 cells cultured in differentiation medium under 1 G conditions normally differentiated to myotubes in an incubation period-dependent manner. However, myogenesis of L6 cells was significantly attenuated under microgravity conditions from Day3 to Day7, although myotubes gradually increased, indicating a retardation of myogenesis under microgravity conditions (Fig. 1). Since myogenesis is regulated by the sequential activation of MRFs, expression levels of myogenesis-related genes were evaluated by real-time RT-PCR. Results showed expressions of *Myod1*, *Myog*, *Myf6*, *Mef2c*, *Des*, and *Ckm* under 1 G conditions were increased in an incubation period-dependent manner (Fig. 2). Among them, *Myod1* expression under 1 G conditions showed a unique transient increase in the early phase (from 12 h to Day1), suggesting initiation of the myogenic process. And expressions of *Myod1*, as well as *Myog* were strongly inhibited under microgravity conditions, suggesting that those inhibitions might have directly affected initiation of myogenesis as in Fig. 1. These significant results suggest that our experimental model would be useful in analysing biological and molecular mechanisms under microgravity conditions.

To clarify the molecular mechanisms of decreased expression of *Myod1* and *Myog*, and their significance in myogenesis under microgravity conditions, L6 cells were first treated with various doses of DNA methylation inhibitor (5-AzaC) for various incubation periods to optimize experimental conditions. As expected, *Myod1* expression increased in a concentration- or a treatment period-dependent manner, although treatment with 10 μΜ of 5-AzaC showed slightly toxic effects. We thus decided to try treatment with 5 μΜ of 5-AzaC for 12 days as an effective condition for the following experiments. Real-time RT-PCR showed that expression levels of *Myod1* and *Myog* seemed to increase gradually in a treatment period-dependent manner, but the transient increase of *Myod1* in the early phase, seen in Fig. 2, disappeared. And differences in expression levels of *Myod1* and *Myog* between 1 G and 10^−3 ^G conditions also disappeared. Furthermore, differences in myotube thickness between 1 G and microgravity conditions completely disappeared in this experimental condition, suggesting that modifying DNA methylation status is essential to regulate myogenesis and expression of *Myod1* and *Myog* under microgravity conditions (Fig. 3d,e). These results suggest that DNA methylation is a key factor in gravity-regulating myogenesis, and possibly a molecular target for treatment of skeletal muscle dysfunctions and Sarcopenia.

UCSC Genome Browser indicated that *MYOD1* gene virtually overlapped with CpG island, indicating very dense CpG sites, and the proximal promoter region actually contained methylated sites (Fig. 4a). In fact, our modified HELP-assay showed that methylated *Myod1* promoter had gradually decreased (PCR product decreased) until Day3, and then increased until Day7. These kinetic changes in DNA methylation status apparently contributed to increased expression of *Myod1* in the initiation process of myogenesis. Furthermore, these kinetic changes in DNA methylation status were attenuated under microgravity conditions, indicating that microgravity regulated both myogenesis and *Myod1* expression by controlling DNA methylation status. The mRNA expressions of *Dnmt1* and *Dnmt3a* gave further evidence that expression levels in DNA methyltransferases might contribute to differences in DNA methylation status under microgravity conditions.

Singh et al. reported that simulated microgravity increased expression levels of *DNMT1*, *DNMT3a*, and *DNMT3b* at 72 h, and reduced them at 7 days in human T-lymphocyte cells. Also, the methylation sensitive-random amplified polymorphic DNA (MS-RAPD) analysis revealed that simulated microgravity exposure resulted in DNA hypomethylation and mutational changes.^27^ In our study, with similar kinetics, increased levels of *Dnmt1* and *Dnmt3a* at 1-5 days and decreased levels at 7 days were observed, but DNA methylation status should have opposite effects: Hypermethylation resulted in decreased expression of *Myod1* and retardation of myogenesis. Effects of *Tdg* kinetics were not consistent with the DNA methylation status. These differences might be due to differences in cell type, culture system, method of methylation detections, or target gene. Although our findings seem consistent, further investigation will be necessary to clarify the results. Other experiments to evaluate promoter activities and mRNA stabilities have also supported the importance of epigenetic regulation of *Myod1* in myogenesis and MRF expression under altered gravity conditions.

**Fig. 5:**
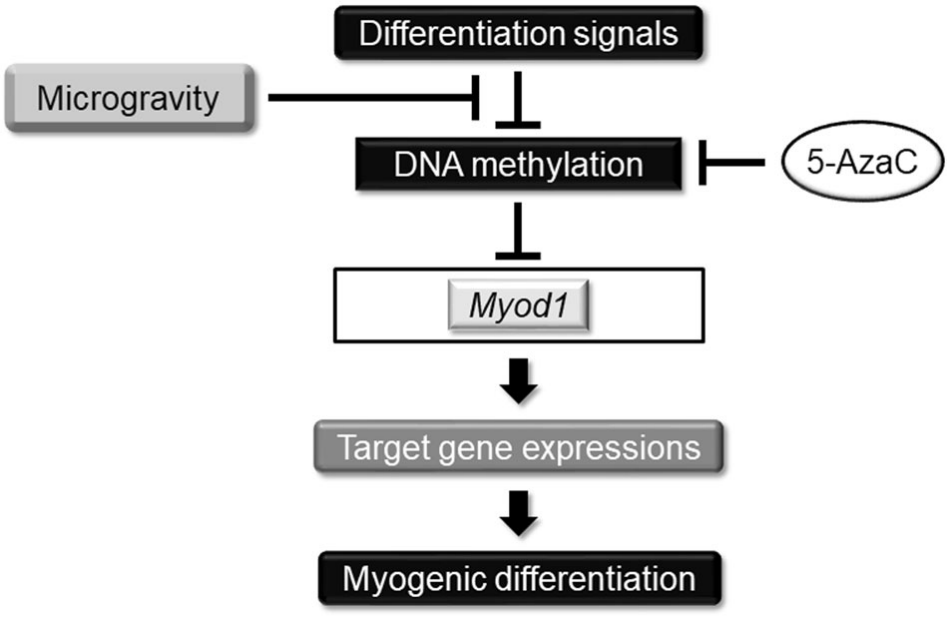
Hypothetical model of regulation of myogenesis. Differentiation signals regulate DNA methylation machinery, and especially, the *Myod1* gene in myoblast cells, resulting in sequential activation of target genes and consecutive myogenic differentiation. Simulated microgravity attenuated this myogenesis by controlling DNA methylation status of *Myod1*. This signal pathway was also controlled using DNA methylation inhibitor, 5-AzaC, treatment

In conclusion, we demonstrated that simulated microgravity attenuated myogenesis by controlling DNA methylation status of *Myod1* (Fig. 5). It is notable that a DNA methylation inhibitor attenuates the inhibitory effects of microgravity on myogenesis, suggesting the inhibitors’ potential as a molecular targeting therapy for muscle atrophy and weakness. Although this study mainly focused on biological viewpoints, further analyses including the influence of external physico-chemical parameters will probably provide new insight into the molecular mechanisms of myogenesis, and also the development of molecular targeting therapy for age-related skeletal muscle dysfunction in the clinical phase.

## Materials and methods

### Cell culture

Rat myoblast cells, L6 cells, were obtained from Health Science Research Resources Bank (Osaka, Japan) and maintained with Dulbecco’s modified Eagle’s minimal essential medium-High glucose (DMEM-H) (NACALAI TESQUE, Inc., Kyoto, Japan) containing 10% fetal bovine serum (FBS; BioWhittaker, Verviers, Belgium), 100 U/ml penicillin, and 0.1 mg/ml streptomycin (Sigma, St. Louis, MO, USA) as previously described.^17^ Cells were cultured with growth medium for 2 days, and then replaced with differentiation medium containing DMEM-H, 2% FBS, 100 U/ml penicillin, and 0.1 mg/ml streptomycin after reaching sub-confluence. Cells were then cultured under normal 1 G or 10^-3^G conditions, using “Gravite^®^” (Space Bio-Laboratories Co., Ltd., Hiroshima, Japan).

For expression analysis, cells were cultured under normal 1 G (group 1 G) or 10^-3^ G conditions (group 10^−3^G) for 0, 12 h, 1, 3, 5, or 7 days. Cells were then harvested and stored at −80˚C until use. L6 cells were also cultured with 5 μM of 5-Azacytidine (5-AzaC, Wako Pure Chemical Industries, Ltd., Osaka, Japan) in growth medium for 12 days, with passage every 4 days. Cells were then seeded on 12.5 cm^2^ culture flasks and cultured in growth medium for 2 days. After medium replacement with differentiation medium, cells were cultured in normal 1 G or 10^−3^ G conditions. Morphological observations and harvesting were performed out at 0, 12 h, 1, 3, 5, and 7 days, and stored at −80 °C until use. Methods were performed in accordance with relevant regulations and guideline.

### Gravite^®^

Microgravity conditions can be produced either by space flight or by free fall; to simulate microgravity, we used a newly developed Gravite® (Space Bio-Laboratories Co., Ltd.), as previously patented (undifferentiated pluripotent stem cell proliferation/differentiation regulation method and system, Patent No. 8034616B2 (US), 2515552 (CA), 1577380 (EPC: GB, FR, DE, IT, SE), ZL02830112.9 (CN), and O731940 (KR), and GRAVITY CONTROLLER, Patent No. 623009 (JP), US9494949B2 (US) and granted in EU). This device produces an environment similar to that of outer space (10^−3^ G) by rotating a sample around two axes, integrating the gravity vector with the temporal axis. This is accomplished by rotation of a chamber at the center of the device, resulting in uniform dispersion of the gravity vector within a spherical volume, with a constant angular velocity. These specific conditions produced a simulated environment of 10^−3^ G in 8 minutes actually measured by gravity acceleration sensor, and it was defined as simulated microgravity (10^−3^ G).^33–35^

### Analysis of myogenesis

To evaluate myogenesis, cells were observed by taking pictures of 10 fields using inverted phase contrast microscope (Eclipse, Nikon, Japan) after incubation under 1 G or 10^−3^ G conditions for indicated periods. The maximum transverse diameters of myotube cells were measured by image processing software ImageJ, and the mean value of 10 fields was calculated.

### RNA preparation and real-time RT-PCR

Total RNA was extracted from frozen cell pellets using the NucleoSpin^®^ RNA II kit (MACHEREY-NAGEL, Düren, Germany) according to manufacturer instructions. Two micrograms of total RNA extracted from each cell line were reverse-transcribed using the High-Capacity cDNA Archive™ Kit (Applied Biosystems, Foster City, CA, USA). A 1/200 dilution of the cDNA was subjected to real-time RT-PCR using primers (final concentration of 200 nM each) and MGB probe (final concentration of 100 nM, the Universal Probe Library: UPL, Roche Diagnostics, Tokyo, Japan) (shown in S1 Table) sets with FastStart Universal Probe Master (ROX) (Roche Diagnostics) for quantitation of gene expressions with *Actb* as an internal housekeeping control. PCR reactions were carried out using 7500 Real-Time PCR System (Applied Biosystems) under the following standard conditions: Three independent measurements were averaged and relative gene expression levels were calculated as a ratio against *Actb* expression for each experiment.

### DNA extraction and methylation analysis

Genomic DNA was isolated from frozen cell pellets using the NucleoSpin^®^ Tissue (MACHEREY-NAGEL) according to manufacturer instructions. Fifty ng of genomic DNA in total 50 μl reaction mixture were digested with methylation-sensitive *Hpa*II at 37 °C for 12 h, and 2 μl of reaction mixture were then subjected to real-time PCR using primer set (shown in S2 Table) to amplify a fragment including CpG sites in *Myod1* promoter region with iTaq^®^ SYBR™ Green Supermix with ROX (BIO-RAD, Hercules, CA, USA). PCR reactions were carried out using 7500 Real-Time PCR System (Applied Biosystems) under the following standard conditions: Three independent measurements were averaged and relative gene expression levels were calculated as a ratio to sample DNA at Set0.

### Plasmid construction and luciferase reporter experiments

The 1.47 kb DNA fragment of the *Myod1* gene (−1264 to +210 from the transcriptional start site at +1) or the 1.55 kb DNA fragment of the *Myog* gene (−1512 to +34 from the transcriptional start site at +1) were amplified by PCR (primer sets are shown in S3 Table) from L6 genomic DNA, and they were subcloned into the luciferase reporter plasmid pGL4.16 (Promega, Madison, WI, USA) (designated pGL4.16-Myod1 Pro1474 or pGL4.16-Myog Pro1546). L6 cells were seeded on culture flasks, and cultured in growth medium for a day. Then reporter constructs were transiently transfected into L6 cells using TransIT^®^-LT1 Transfection Reagent (TaKaRa Bio, Inc., Shiga, Japan). The renilla luciferase vector (pRL-SV40, Promega) was used as a transfection efficiency control. After incubation for a day, growth medium was replaced with differentiation medium and incubated for one more day under 1 G or 10^-3^G conditions. Luciferase luminescence was measured using a single-sample luminometer, the Biolumat LB 9505 (BERTHOLD TECHNOLOGIES GmbH & Co. KG, Bad Wildbad, Germany) with the Dual-Luciferase Reporter Assay System (Promega). Promoter activities were calculated as the ratio of firefly to renilla luciferase readings, and the averages of at least three independent experiments were calculated. Methods were approved by a Hiroshima University Gene Recombination Experiment Safety Committee.

### Analyses of mRNA stabilities

L6 cells were seeded on culture flasks, and cultured in growth medium. After 2 days, growth medium was replaced with differentiation medium and incubated for another day. After adding 5 μg/ml of actinomycin D (NACALAI TESQUE) to the medium, cells were cultured under 1 G or 10^−3^ G conditions for the indicated periods (0, 15, 30, 45, 60, or 120 min). Total RNA was prepared from each harvested cell, and gene expressions of *Myod1*, *Myog*, and *Actb* were analysed using real-time RT-PCR method as mentioned above.

### Statistical analysis

All statistical tests were performed using the StatView^®^ version 5.0 software (SAS Institute Inc., NC, USA) and Microsoft^®^ Excel^®^ for Mac version 12.3.6. Student’s *t*-test was used to determine *P*-values (*P < 0.05, **P < 0.01).

### Data availability

All relevant data are available from the corresponding author.

## Electronic supplementary material

Supplementary Information
