# Publisher Correction: Simulated microgravity attenuates myogenic differentiation via epigenetic regulations

**DOI:** 10.1038/s41526-018-0047-y

**Published:** 2018-07-18

**Authors:** Takuma Furukawa, Keiji Tanimoto, Takahiro Fukazawa, Takeshi Imura, Yumi Kawahara, Louis Yuge

**Affiliations:** 1 Division of Bio-Environmental Adaptation Sciences, Graduate School of Biomedical and Health Sciences, Hiroshima University, Hiroshima, Japan; 2 Department of Radiation Medicine, Research Institute for Radiation Biology and Medicine, Hiroshima University, Hiroshima, Japan; 3 Natural Science Center for Basic Research and Development, Hiroshima University, Hiroshima, Japan; 4 Space Bio-Laboratories Co., Ltd, Hiroshima, Japan

**Correction to**: *npj Microgravity* 10.1038/s41526-018-0045-0, Published online 23 May 2018

The original version of this Article contained an error in Fig. 4. Fig. 4B was partly cropped, which obscured some of the sequence information. The correct version of Fig 4B appears below as Fig. 1, which replaces the previous incorrect version that appears as Fig. 2 below. This has been corrected in both the PDF and HTML versions of the Article.

**Fig. 1:**
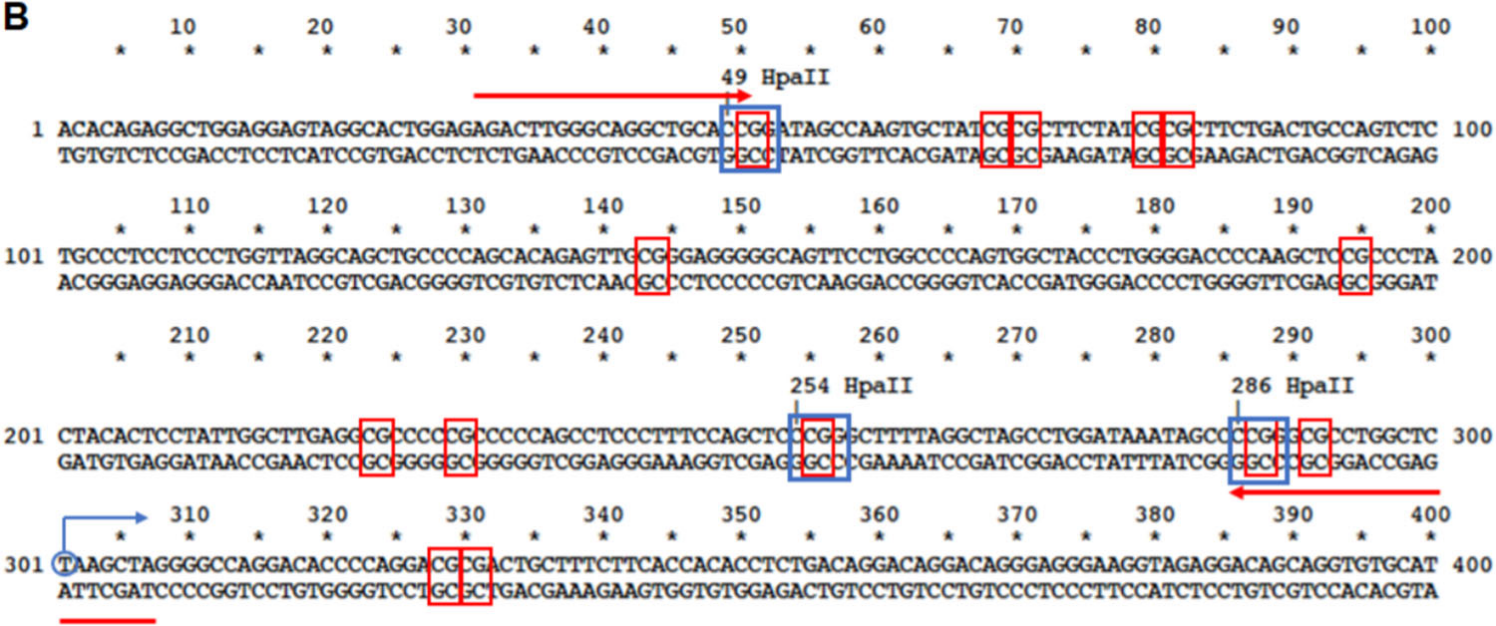
▓

