# Supplementary material for: Simulated microgravity attenuates myogenic differentiation via epigenetic regulations
Source: NPJ Microgravity. 2018 May 23;4:11. doi: 10.1038/s41526-018-0045-0 (PMC5966377; doi:10.1038/s41526-018-0045-0)
Supplement: Supplementary file 1 — Supplementary Information [file 41526_2018_45_MOESM1_ESM.pdf]

Supplementary Figures

Figure S1

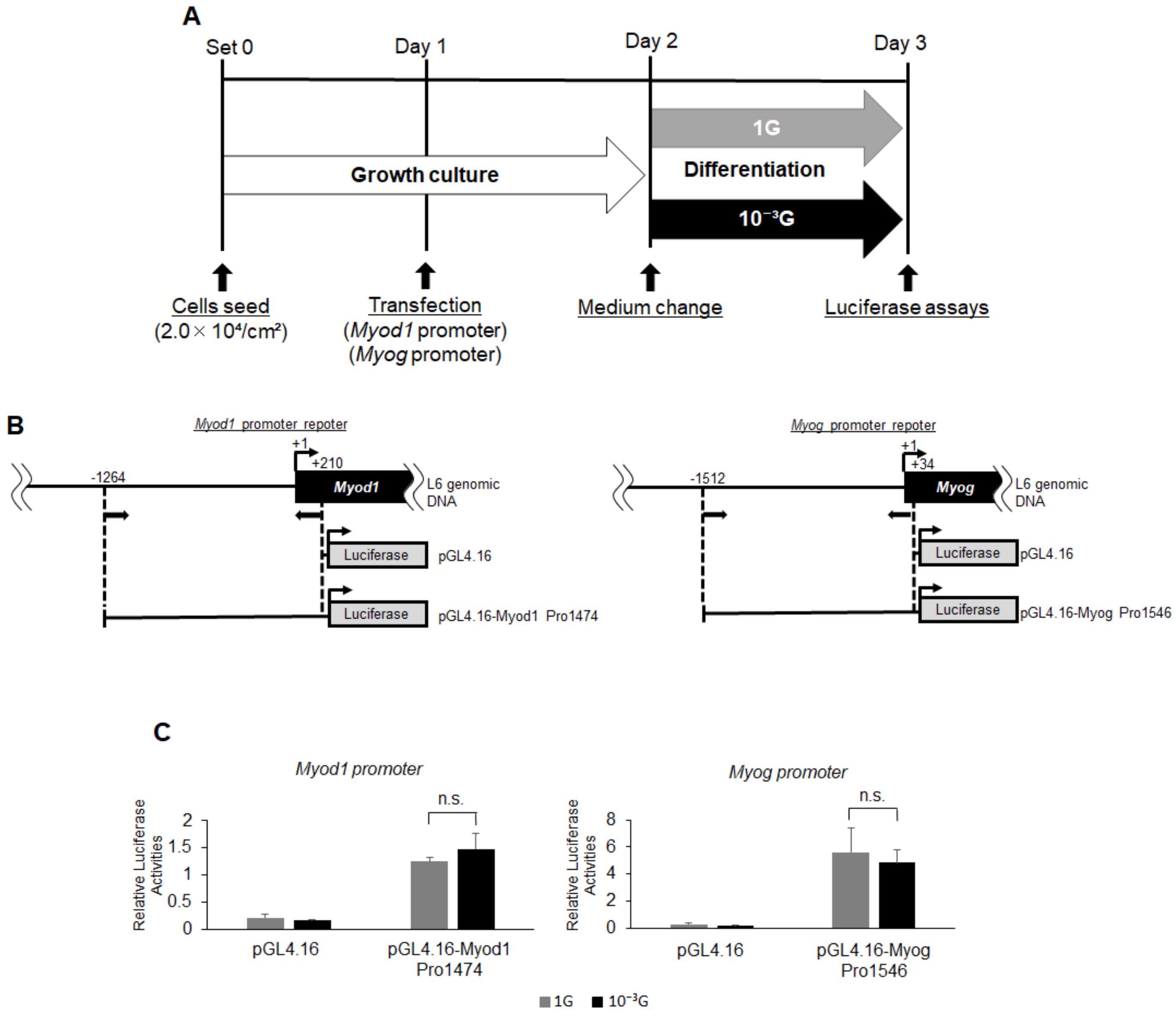

Figure S2

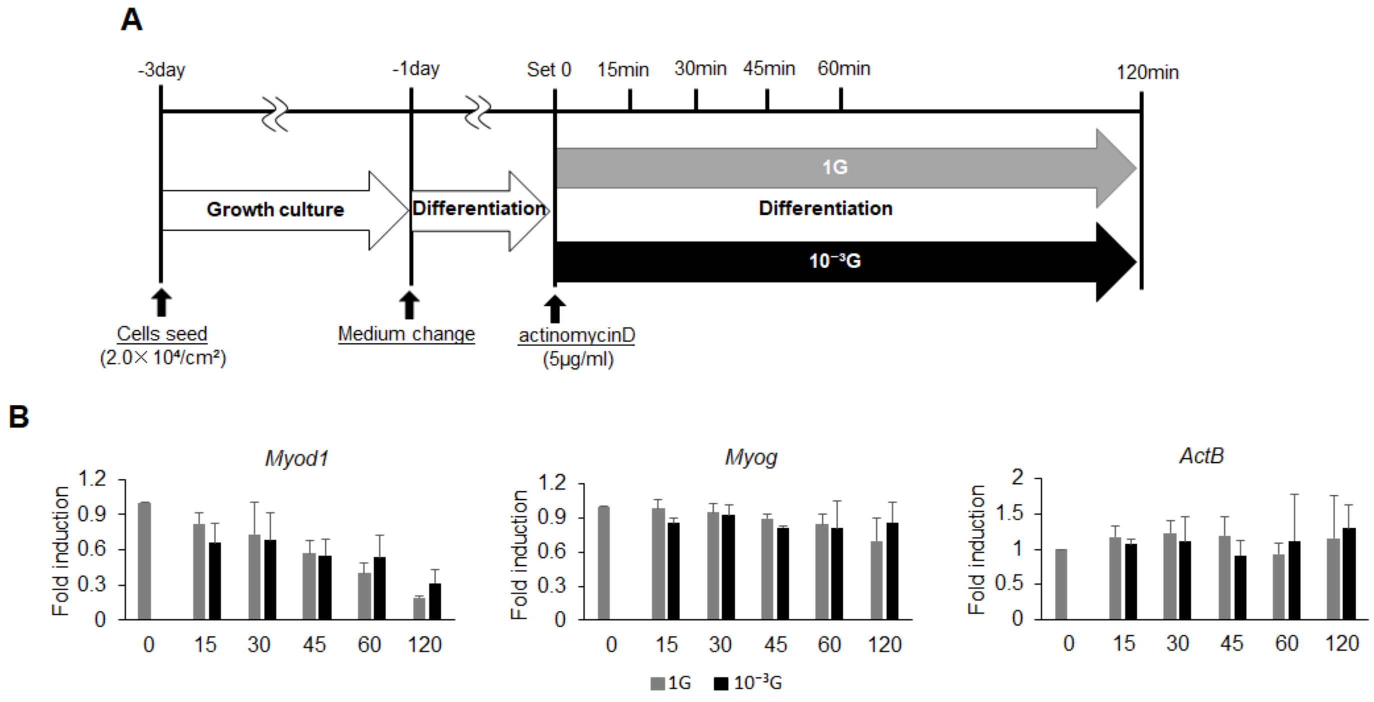
A
-3day
-1day
Set 0
15min
30min
45min
60min
120min
Growth culture
Differentiation
1G
Differentiation
10⁻³G
Cells seed
(2.0× 10⁴/cm²)
Medium change
actinomycinD
(5μg/ml)
B
Myod1
Myog
ActB
Fold induction
0
15
30
45
60
120
1G
10⁻³G

Supplementary Tables

S1 Table. Primer and probe sets for real-time RT-PCR

Myod1	Forward: 5’-CCCTGTTGTTTGTGGAGACA-3’	
	Reverse: 5’-CTGTGGGAAAGAGTGGGTGT-3’	
	Universal Probe Library: #50	
Myog	Forward: 5’-CTACAGGCCTTGCTCAGCTC-3’	
	Reverse: 5’-TGGAGTTGCATTCACTGG-3’	
	Universal Probe Library: #63	
Myf6	Forward: 5’-GGGCCTGGTGATAACTGCT-3’	
	Reverse: 5’-AGAAAGGCGCTGAAGACTG-3’	
	Universal Probe Library: #12	
Des	Forward: 5’-GCCTTGGATGTGGAGATTG-3’	
	Reverse: 5’-TGGATCGGAAGGTTGATCC-3’	
	Universal Probe Library: #120	
Ckm	Forward: 5’-CTGCCCTGATGTATCAAACG-3’	
	Reverse: 5’-GAACTTGTTGTGGGTGTTGC-3’	
	Universal Probe Library: #20	

Myf5 Forward: 5’-ACAGCAAAGACAGACACTCAGG-3’

Reverse: 5’-TGTGTCCTGAAGAGCCAACTC-3’

Universal Probe Library: #21

Mef2c Forward: 5’-GGCTTCAATACTGCCAGTGC-3’

Reverse: 5’-TGAGATAAATGAGTGCTAGTGCAA-3’

Universal Probe Library: #74

Pax3 Forward: 5’-TTACTGAGGCCCGAGTGC-3’

Reverse: 5’-CATCAGTTGATTGGCTCCAG-3’

Universal Probe Library: #69

Pax7 Forward: 5’-ACCACTACCCGGACATCTA-3’

Reverse: 5’-CACGACGGTTACTGAACCAG-3’

Universal Probe Library: #17

Dnmt1 Forward: 5’-AACGGTGTTGTCTACCGACTG-3’

Reverse: 5’-GCTGGCCATTTTGATGTTG -3’

Universal Probe Library: #16

*Dnmt3a*Forward: 5’-AACTGAGACCCCACCAGAAG-3’

Reverse: 5’-TGGTCTGCTTCTGTTCTTTGC-3’

Universal Probe Library: #66

*Dnmt3b*Forward: 5’-GATGCCAGGACTCCCTCTG-3’

Reverse: 5’-TTCTGGGGGAGGTTCTTTG-3’

Universal Probe Library: #64

Mbd2 Forward: 5’-TCAGCGGATGAATGAACAAC-3’

Reverse: 5’-TCTGATGCGCTAAGTCCTTG-3’

Universal Probe Library: #7

Tdg Forward: 5’-GCTCCGAACATGGCAGAC-3’

Reverse: 5’-CCTTCTCTTTGGAGCCTCTG-3’

Universal Probe Library: #56

Actb Forward: 5’-CCCGCGAGTACAACCTCCT-3’

Reverse: 5’-CGTCATCCATGGCGAACT-3’

Universal Probe Library: #17

S2 Table. Primer sets for real-time PCR

Myod1 promoter region	Forward: 5’-AGACTTGGGCAGGCTGCACC-3’
	Reverse: 5’-TAGCTTAGAGCCAGGCGCCC-3’

S3 Table. Primer sets for promoter cloning

Myod1 promoter	Forward: 5’-GGTACCTCCCCAGTGAACCTGCTG-3’	
	Reverse: 5’-GTCCCAGTTCTGGGTCCTGC-3’	
Myog promoter	Forward: 5’-GGTACCTGTTTCCTCCTTCAAAAGACAG-3’	
	Reverse: 5’-CAGGTCGGAAAAGACTTGTTC-3’	

Supplementary Information

S1 Fig. Promoter activities of *Myod1* and *Myog* genes under microgravity conditions. (A) Schematic representation of experimental time course is shown. L6 cells were transfected with *Myod1* or *Myog* promoter reporters (designated pGL4.16-Myod1 Pro1474 or pGL4.16-Myog Pro1546) and incubated for 1 day. Growth medium was then replaced with differentiation medium and incubated for one more day under 1G or 10^{-3}G conditions. (B) Schematic structures of promoter regions and luciferase reporters of *Myod1 and Myog* genes. (C) Relative luciferase reporter activities were analyzedusing dual-luciferase assays and then calculated as the ratio of firefly to renilla luciferase readings. Columns show the mean of three independent experiments; bars, SD. (n = 3)

S2 Fig. mRNA stabilities of *Myod1* and *Myog* genes under microgravity conditions. (A) Schematic representation of experimental time course is shown. L6 cells were cultured for 2 days in growth medium. Medium was then replaced with differentiation medium and incubated for one more day. After adding 5 μg/ml of actinomycin D, cells were cultured under 1G or 10^{-3}G conditions for 0, 15, 30 45, 60 or 120 min. (B) Expression levels of *Myod1*, *Myog*, and *Actb* genes were evaluated by real-time RT-PCR. Three independent measurements were averaged and relative gene expression levels were calculated as the ratio against *Actb* expression for each experiment. Columns show the mean of three independent experiments; bars, SD. (n = 3)
